# Health-Related Quality of Life, Blood Pressure, and Biochemical and Anthropometric Profile in Vegetarians and Nonvegetarians

**DOI:** 10.1155/2020/3629742

**Published:** 2020-07-07

**Authors:** Jacksaint Saintila, Tabita E. Lozano López, Percy G. Ruiz Mamani, Michael White, Salomón Huancahuire-Vega

**Affiliations:** ^1^Departamento de Nutrición, Escuela de Nutrición Humana, Facultad de Ciencias de La Salud, Universidad Peruana Unión, Lima 15, Peru; ^2^Universidad Privada San Juan Bautista, Lima, Peru; ^3^Dirección General de Investigación, Universidad Peruana Unión, Lima 15, Peru; ^4^Departamento de Ciencias Básicas, Facultad de Ciencias de La Salud, Escuela de Medicina Humana, Universidad Peruana Unión (UPeU), Lima 15, Peru

## Abstract

Several studies have been carried out which mainly focus on the analysis of the lipid profile in vegetarians and nonvegetarians. However, few studies have been undertaken in this population oriented to quality of life and health. This study aimed to compare health-related quality of life, blood pressure, and biochemical and anthropometric profile in vegetarians and nonvegetarians. The study included 149 participants out of an initial sample of 162: 62 vegetarians and 87 nonvegetarians. Health-related quality of life was assessed with the SF-12 Health Questionnaire version 2 and was related with the lipid profile, glucose, blood pressure, anthropometric measures, and sociodemographic characteristics. Vegetarians presented better Body Mass Index (BMI) and waist circumference (WC), as well as higher LDL levels. No significant differences in HDL and TG concentrations were found. Serum glucose concentrations were significantly lower among vegetarians. Nonvegetarian males had higher diastolic pressure levels. Vegetarian women had significantly higher levels of systolic pressure. As for the physical health and mental health components of quality of life, no significant differences were found in vegetarian and nonvegetarian women and men. In conclusion, vegetarians presented a better anthropometric profile, lower glycaemia, and higher LDL levels but no significant differences in health-related quality of life compared with nonvegetarians.

## 1. Introduction

In recent decades, turning to vegetarianism has become a popular trend among global populations [1]. There are many reasons why people opt for a vegetarian diet; they range from an attempt to seek improved physical and mental well-being, where there may be evidence of reduced risk factors for chronic noncommunicable diseases and mortality risks, to a desire to care for the environment and compassion for animals [2].

The vegetarian diet is generally characterized by abundant fruits, vegetables, whole grains, nuts, legumes, and seeds, although sometimes foods of animal origin such as eggs and dairy products are introduced [3]. A well-planned vegetarian diet in which minimally refined foods predominate can provide adequate food and nutrition [2] at any stage of life, including pregnancy, lactation, infancy, childhood, and adolescence, and in sports [3].

However, irregular consumption of plant-based foods can contribute to serious problems in a person's lipid profile, increasing metabolic risk factors such as increased concentration of triglycerides (TG), low density lipoproteins (LDL), increased glucose concentration, high blood pressure, high Body Mass Index (BMI), and high Waist Circumference (CC) [4]. Furthermore, it is one of the main causes of the appearance of cardiovascular diseases, type II diabetes mellitus [5], and different types of cancer [3, 6]. In contrast, adequate and optimal daily intake of minimally refined plant foods can help improve LDL and TG concentrations [4], prevent hypertension [7], and maintain WC [8] and Body Mass Index (BMI) within normal ranges, including HDL levels in an optimal range [9].

The scientific evidence relating vegetarian diets to a better lipid profile [10], a lower prevalence of obesity, hypertension, hypercholesterolemia, type 2 diabetes mellitus, and a lower prevalence of mortality from ischemic heart disease and stroke is based on their nutritional composition [9]. In contrast, diets based on the consumption of meat and its derivatives play a crucial role in the increase of these chronic noncommunicable diseases because they are characterized by high caloric density, excess saturated fat, and simple sugar [11]. Consumption of red meat has been associated with the development of arterial thrombosis, cardiac ischemia, high blood pressure, and acute thrombotic infarction, among others, due to increased platelet aggregation [12]. Additionally, the consumption of saturated fats from meat products can significantly increase insulin secretion, which could lead to insulin resistance and the appearance of type II diabetes mellitus [13].

Furthermore, the vegetarian diet plays a fundamental role in improving the psychological dimension of quality of life [14]. Scientific evidence suggests that restricting foods, based on meat and its derivatives, fish, and poultry, could improve some indicators of people's mental health status [15]. Additionally, based on WHO recommendations, it contributes to better mental health, lower incidence of depression and anxiety, greater life satisfaction, and improved emotional well-being [16–18]. These benefits of the quality of life aspects could be due to the presence of vitamin C, folic acid, and carotenoids. These nutrients present in fruits and vegetables act as cofactors of dopamine and other neurotransmitters [19].

Previous research has focused on comparing biochemical and anthropometric profiles in vegetarians and nonvegetarians; however, few have focused on comparing quality of life in the two population groups. This study aims to compare health-related quality of life, blood pressure, and biochemical and anthropometric profiles in vegetarians and nonvegetarians.

## 2. Materials and Methods

### 2.1. Design, Type of Research, and Participants

This was a cross-sectional study. Through an intentional nonprobabilistic sampling, 169 participants were recruited from vegetarian restaurants, schools, and a university belonging to a religious community. Twenty participants were excluded because they did not correctly complete all the items in the sociodemographic and anthropometric sections. Excluded were those under 17 and over 59 years of age, those who did not sign the informed consent, those who underwent medical treatment, and those who had chronic noncommunicable diseases such as diabetes, cardiovascular disease, dyslipidemia, and hypertension. The study complied with the ethical standards of data collection (confidentiality and freedom of participation) and received the approval of the Health Science Research Ethics Committee of the Universidad Peruana Unión (N°00124-2019/UPeU/FCS/CIISA).

### 2.2. Quality of Life SF12v2

To evaluate the participants' quality of life, the SF-12 Health Questionnaire version 2 was used. The Cronbach coefficient *α* was ≥0.7 (23). This questionnaire is the most widely used on a global level and is very useful for assessing and monitoring the state of health in the population in general or in some population groups that have a certain disease. In addition, it allows data to be obtained regarding those secondary factors that can significantly influence people's quality of life. It consists of 12 questions with different answer alternatives and measures 8 aspects (dimensions) of quality of life: physical function, social function, physical role, mental health, vitality, emotional role, body pain, and general health. By combining the scores of each of the dimensions, the questionnaire is classified into summary scales of two synthetic components: physical health components (PHC) and mental health components (MHC).

### 2.3. Sociodemographic Characteristics and Blood Pressure

A record card was used to obtain information on sociodemographic background (type of diet, level of education, marital status, and origin). Participants' blood pressure measurements were obtained using the Omron HEM-747IC Automatic Blood Pressure Monitor (Omron Healthcare, Inc., Vernon Hills, IL), and the mean value used for the analysis was evaluated with the assistance of a nurse hired by the study investigators. A systolic blood pressure greater than or equal to 140 mmHg and a diastolic blood pressure greater than or equal to 90 mmHg in adults indicate hypertension, according to clinical guidelines for the management of hypertension in Peru [20, 21].

### 2.4. Anthropometric Measurements

The determinations of the anthropometric measurements were carried out in the Nutritional Consultation Room of the Universidad Peruana Unión. Weight and height were measured using a calibrated SECA 700 mechanical column scale with a capacity of 220 kg and a measurement range of 60 to 200 cm (SECA®, Hamburg, Germany). The BMI was calculated according to the parameters established by the WHO, using the Quetelet index, and was classified as follows: underweight, ≤18.5; normal weight, ≥18.5–≤24.9 kg/m^2^; overweight, between 25.0 and 29.9 kg/m^2^; obesity grade 1, between ≥30 and ≤34.9 kg/m^2^; obesity grade 2, between ≥35 and ≤39.9 kg/m^2^ and Obesity 3, ≥40 kg/m^2^. The WC was determined through a self-retracting metallic steel tape measure of the Cescorf brand (Cescorf Equipamentos Para Esporte Ltda—Epp, Brazil). Abdominal obesity was considered for a WC ≥ 93 cm in men and ≥79 cm in Peruvian adult women.

### 2.5. Lipid Profile and Blood Glucose Concentration

Blood samples (5 ml) were drawn during the first two hours of the morning, after 12 hours of fasting according to standard procedures for blood samples. To determine the lipid profile, commercial Colestat Enzyme AA kits from Wiener lab were used. Blood glucose was determined using colorimetric enzymatic methods performed semiautomatically. The participants' lipid profile was classified as follows: high levels of LDL (LDL ≥ 160 mg/dL), low levels of HDL (HDL-c < 40 mg/dL in men and <50 mg/dL in women), and hypertriglyceridemia (TG ≥ 200 mg/dL). Fasting glucose concentration ≥126 mg/dL was considered hyperglycemia. Blood collection and serum processing were performed by a Certified Medical Technologist from the Microbiology Laboratory of the Universidad Peruana Unión.

### 2.6. Statistical Analysis

The data obtained was entered into a Microsoft Excel 2013 spreadsheet. The statistical analyses were performed using the IBM SPSS version 24.0 program. Student's *t*-test was used for the mean difference between groups and the chi-square test for the contrast of proportions in the sociodemographic characteristics. A significance level of 0.05 was considered.

## 3. Results

149 participants gave their consent and were included to participate in the study. Participants were divided into two groups according to their diet: 62 vegetarians and 87 nonvegetarians.  Table 1 shows the sociodemographic characteristics of the participants. The vegetarians and nonvegetarians who were between 36 and 59 years old represented 46.8% and 41.4% of the sample, respectively. Females predominated with a representation of 69.4% and 65.5% of the sample in vegetarians and nonvegetarians, respectively. Both vegetarians and nonvegetarians had nearly equal proportions of being married and single. In terms of education level, the highest proportion (62.9% and 24.2%) of vegetarians were more likely to have a college degree and a graduate degree, respectively, compared to nonvegetarians (51.7% and 19.5%).

As shown in Table 2, vegetarian males had a lower average weight (66.28 kg) than nonvegetarian males (74.97 kg), and there was a statistically significant difference between the two groups (*t* = 3.13, *P*=0.003). The height was higher (1.72 mts) in the vegetarian males than in the nonvegetarian males (1.68 mts); the significant difference had a value of *P*=0.010. No significant difference was found in the size of vegetarian and nonvegetarian women. It was observed that the BMI of vegetarian males was lower (22.41 kg/m^2^) than nonvegetarian males (26.63 kg/m^2^) with a significant degree of difference (*t* = −5.16, *P* ≤ 0.001). The data do not show significant differences between the BMI of vegetarian and nonvegetarian women. In addition, the WC of male vegetarians was significantly lower (80.13 cm) (*t* = −3.86, *P* ≤ 0.001) compared to nonvegetarians (89.79 cm); however, it was not considered abdominal obesity (WC ≥ 93). The findings showed no abdominal obesity or significant difference in the ranges of WC in vegetarian and nonvegetarian women. On the other hand, vegetarian women had a higher LDL level (115.65 mg/dL) compared to nonvegetarian women (100.53 mg/dL). No significant differences were observed in vegetarian and nonvegetarian men and women in HDL and TG concentrations. The means for all the lipid profile indicators were within normal values. Vegetarian males had significantly lower blood glucose concentration (74.47 mg/dL) than nonvegetarian males (81.83 mg/dL) (*t* = −2.05, *P*=0.046), while in nonvegetarian females, no significant difference was found in the two dietary patterns for blood glucose, although they were within normal values. Vegetarian women had higher average SBP levels (105.44 mmHg) than nonvegetarian women (99.84 mmHg). These findings were significant (*t* = 2.43, *P*=0.017). Vegetarian males had significantly lower SBP levels (71.11 mmHg) than nonvegetarian males (77.50 mmHg), (*t* = −2.78, *P*=0.008). The mean of the blood pressure measurements did not show values that indicate hypertension. As for the physical health and mental health components of quality of life, no significant differences were found in both female and male vegetarians and nonvegetarians.

## 4. Discussion

The type of diet has an influence on the quality of life and the lipid profile in people; it is believed that dietary patterns, in which adequate consumption of fruits, vegetables, whole grains, nuts, legumes, and seeds predominates, play an important role in improving the quality of life, as well as reducing metabolic risk factors [2, 3].

The study found a higher proportion of adults in the vegetarian population (Table 1); these results are consistent with those found in other research [22, 23]. Although these differences were not significant, being in the adult age range, a conscious need to take care of their health through healthy eating may arise in people in order to prevent chronic diseases. Also, in relation to educational level, there is a higher proportion of people with university and postgraduate degrees in the vegetarian population. These characteristics have been reported in previous studies [24–26]. It is possible that education is significantly associated with the intake of plant-based foods such as fruits and vegetables and, at the same time, could be considered a strong predictor in the choice of a healthy dietary pattern. A high degree of education can provide individuals with a resource and tool for nutrition education and health promotion in order to improve eating habits and establish healthier behavior [22].

Regarding the quality of life of the participants, the data have not demonstrated significant differences in the mental health component (Table 2). A similar study has shown that the mental health scores of vegetarians and semivegetarians were significantly lower than nonvegetarians [23]. Also, related evidence was reported where the vegetarian diet is associated with an increased risk of mental disorders [27, 28]. In these studies, it is not possible to establish a relationship based on cross-sectional data whether or not a dietary pattern is responsible for these differences in mental health. Furthermore, there is no evidence of the causal role of vegetarian diets in the etiology of mental disorders. On the other hand, there are studies that show that a higher intake of fruits and vegetables is beneficial for mental health [29]. Although the biological pathways of the effects of plant-based food intake on mental health remain unknown, there are several mechanisms that could explain this link: complex carbohydrates, folic acid, vitamin B6, and certain antioxidants and minerals may have some positive impact on mental health because they are responsible for the synthesis of neurotransmitters, acting against oxidative stress and inflammation [30]. Similarly, the antioxidant properties and biomodulatory effects of polyphenols on cell signaling pathways related to neuronal plasticity and neuronal system stability may act against psychiatric disorders [31].

As for the physical health component, no significant differences were found; however, it was evident that vegetarians had a better score (Table 2). Similar findings were reported by Baines et al. [24]. Additionally, it was observed that vegetarian diets have protective value against various physical and chronic ailments, such as type II diabetes mellitus, cardiovascular disease, high blood pressure, and different types of cancer [3, 13, 32, 33]. These protective actions are evidenced by the content of dietary fiber, phenols, and antioxidants. The presence of free radicals in the body in excess is one of the main causes of the diseases already mentioned. Antioxidants and other bioactive elements in vegetarian diets protect the body against harmful free radicals, reducing the risk of oxidative stress and inflammation. They also inhibit malignant transformation and cancer mutations and reduce the proliferation of cancer cells [34].

Vegetarians were found to have lower BMIs and WCs than nonvegetarians (Table 2). The relationship between BMI, WC, and plant-based dietary patterns is well documented in the scientific literature; some prospective studies [9, 35] give evidence that pesco-vegetarians, lacto-ovo vegetarians, and vegans have a lower BMI compared to nonvegetarians. In addition, other studies [22, 24, 28] have shown that the BMI in the vegetarian population is within the normal parameters compared to the population that follows a pattern of eating products of animal origin. Although the mechanisms of action of the components of vegetarian diets in relation to weight and BMI reduction are not so clear, it is assumed that vegetarians consume fewer total calories, less saturated fat, a low intake of animal protein, and a high intake of dietary fiber, which could be of great benefit in controlling caloric intake by producing a feeling of satiety [12]. The intake of animal protein and the increase in serum concentration of insulin growth factor type 1 (IGF-1) levels would be responsible for the increase in BMI in nonvegetarians [36]. In addition, IGF-1 is an important factor in the differentiation and maturation of fat cells [37]. With respect to WC in the vegetarian population, the risk of abdominal obesity is very low, which could be due to a lower consumption of animal fat and a higher consumption of bioactive elements (lycopene, resveratrol, among others) from vegetables [38].

In this study, vegetarian women were found to have higher LDL levels than nonvegetarian women (Table 2). However, these values were within the normal range. In contrast, several studies found more favorable LDL levels compared to nonvegetarians [39, 40]. However, the atherogenic activity and endothelial lesions promoted by this type of oxidized cholesterol would be minimized by the high content of antioxidants present in the vegetarian diet. The lack of regular physical activity and a possible high consumption of trans fatty acids, substances widely used in the manufacture of refined carbohydrates, could be some of the causes of the higher LDL levels in vegetarians. Furthermore, beyond the possible protective effects of plant-based diets, it is important to consider that those vegetarians who have been following this regimen for less than 5 years may have similar LDL values compared to nonvegetarians; the beneficial results of a healthy lipid profile would be obtained, if there is permanent and regular long-term monitoring of the vegetarian diet. Although, theoretically, plant-based diets are closely linked with the healthy diet and quality of life benchmark [41], it is nevertheless likely that some people following vegetarian diets actually have an unhealthy eating pattern [42].

As for the HDL and TG levels, the results did not differ significantly (Table 2). Kim et al. [43] also found no significant difference in HDL and TG cholesterol concentrations between vegetarians and nonvegetarians. However, other research has shown that vegetarians had higher HDL levels and lower TG concentrations than nonvegetarians [39, 44]. These statistical differences could be due to the quality of the vegetarian diet, which provides a greater amount of fruits, vegetables, whole grains, nuts, and soy, foods which are sources of phytochemicals such as phytosterols and other bioactive compounds that are associated with a reduced lipid profile, including TG, which in turn protect the body by reducing the prethrombic and inflammatory state and improve endothelial function [1, 12]. Scientific evidence has suggested that phytochemicals play an important role in lipid metabolism by improving plasma concentrations. In fact, some phytochemicals bind to peroxisome proliferator-activated receptors that regulate lipid metabolism and promote absorption, utilization, and catabolism of fatty acids through positive regulation of genes involved in transport and beta oxidation of peroxisomal and mitochondrial fatty acids [45].

Blood glucose concentration was significantly lower in vegetarian than nonvegetarian males. Similar findings were found in other studies [8, 32] where vegetarians were observed to have significantly lower serum fasting glucose concentration and higher insulin sensitivity compared to nonvegetarians. Also, Valachovicová et al. [32] have shown significantly lower insulin resistance index (HOMA-IR) values compared to nonvegetarians. Other studies [9, 38, 43] have shown that plant-based diets have a positive action in controlling serum glucose concentration. Low glucose concentrations are an important factor in reducing the risk of metabolic syndrome in the vegetarian population. The proposed reasons why vegetarian diets are associated with a decrease in glucose levels [26] include, in particular, dietary fiber present in whole grains, beans, nuts, and legumes. Dietary fiber, in addition to reducing the glycemic index of carbohydrates, improves glycemic control by increasing the excretion of bile acids and the production of short-chain fatty acids through bacterial fermentation [46]. It is worth mentioning that, in this research study, both glucose and BMI values were significantly lower in vegetarian compared to nonvegetarian males (Table 2). High BMI values would imply an important risk of continuous increase in glucose concentration, while a normal BMI implies a positive effect on glucose concentrations and combines a protective action against diabetes mellitus. The higher the BMI, the greater the risk of developing diabetes [9].

Vegetarian women had higher SBP levels than nonvegetarian women, while vegetarian men had the highest DBP levels compared to nonvegetarians. In contrast, other studies found that vegans and lacto-ovo vegetarians had lower diastolic and systolic blood pressure values than nonvegetarians [8, 13]. In addition to the absence of meat, the intake of specific foods, such as fruits, vegetables, and legumes appear to have a protective effect on lowering blood pressure levels because these foods contain phytosterols, monounsaturated, and polyunsaturated fats [12], which significantly lower serum cholesterol concentrations, which can be a risk factor for high blood pressure [38]. Although the protective actions of vegetarian diets on blood pressure are not very clear, there is evidence that the intake of essential nutrients such as soy protein, dietary fiber, potassium, calcium, magnesium, phosphorus, and antioxidants such as vitamins C and E, act effectively in lowering blood pressure levels [47, 48]. In this study, vegetarians had higher LDL levels than nonvegetarians. The common possible factor that could explain the high LDL concentration and higher blood pressure levels in vegetarians would be the intake of refined carbohydrates, since their excessive consumption is associated with greater adverse effects not only on the lipid profile, but also on blood pressure [49]. On the other hand, although blood pressure levels were higher in vegetarians, it is interesting that they do not have a higher BMI than nonvegetarians, since obesity is the main risk factor related to hypertension [48]. Even so, it should be mentioned that data showing significant differences in blood pressure between vegetarians and nonvegetarians should not always be fully explained by BMI. Generally, compared to meat-based diets, vegetarian diets may contain less sodium concentration; however, no clear difference has been determined [50].

This study has some limitations. The results and conclusions cannot be generalized because the sample was selected through nonprobabilistic methods, in which random selection is not considered. The identification and recruitment of participants were a difficult process because they are not located in the same place. Although the recruitment of participants was done in restaurants, schools, and a university belonging to a religious community, this does not necessarily imply that all participants belong to this religious community. In addition, this study did not measure dietary habits and physical activity, so future studies that include these variables are recommended, as they could influence quality of life, blood pressure, and biochemical and anthropometric profiles.

## 5. Conclusions

No significant differences were found in the components of physical and mental health. In terms of anthropometric data, results have shown that vegetarians had better levels of weight, height, BMI, and waist circumference. Regarding the concentrations of biochemical values, the results have shown that vegetarians had higher levels of LDL concentration than nonvegetarians. The results showed no significant differences in HDL and TG concentrations. Serum glucose concentrations were significantly lower among vegetarians. Nonvegetarian males had higher diastolic blood pressure levels compared to vegetarians. Vegetarian women had significantly higher levels of systolic blood pressure than nonvegetarian women. These results suggest that the exclusion of meat alone from a diet does not necessarily reflect a balanced eating pattern, which would depend on a number of factors, such as abundant consumption of minimally refined plant-based foods, water consumption, and regular physical activity. While vegetarianism is a healthy option, careful planning is necessary to ensure that it is well-balanced.

## Figures and Tables

**Table 1:** Sociodemographic characteristics of vegetarians and nonvegetarians.

	Vegetarians		Nonvegetarians		*χ* ^2^	*P* ^*∗∗*^
	*n*	%	*n*	%		
Age						
≤25	20	32.3	18	20.7	5,515	0.063
26–35	13	21.0	33	37.9		
36+	29	46.8	36	41.4		

Sex						
Female	43	69.4	57	65.5	0,242	0.623
Male	19	30.6	30	34.5		

Origin						
Coast	29	46.8	45	51.7	13,775	0.003^*∗∗∗*^
Jungle	14	22.6	27	31.0		
Sierra	5	8.1	12	13.8		
Foreign	14	22.6	3	3.4		

Marital status						
Single	32	51.6	45	51.7	0,000	0.989
Married	30	48.4	42	48.3		

Level of education						
Basic education	2	3.2	1	1.1	7,709	0.052
Technical school	6	9.7	24	27.6		
Undergraduate degree	39	62.9	45	51.7		
Graduate degree	15	24.2	17	19.5		

Occupation						
Nonacademic staff	13	21.0	37	42.5	12,479	0.002^*∗∗∗*^
University professor	27	43.5	38	43.7		
University student	22	35.5	12	13.8		

^*∗*^Vegetarians are those who do not consume meat, fish, or poultry and nonvegetarians are those who do eat such foods. ^*∗∗*^*P* value. The Chi-squared (*χ*^2^) test was used to evaluate the degree of significance for the sociodemographic data and the type of diet. *P* represents the probability that the dietary pattern is associated with the sociodemographic data. ^*∗∗∗*^Statistically significant.

**Table 2:** Comparative analysis of anthropometric, biochemical, and quality of life data for vegetarians and nonvegetarians by gender.

Variables	Women						Men					
	Vegetarians		Nonvegetarians		*t*	*P*	Vegetarians		Nonvegetarians		*t*	*P*
	Mean	SD	Mean	SD			Mean	SD	Mean	SD		
Weight (kg)	57.15	7.90	58.78	9.19	−0.93	0.354	66.28	7.94	74.97	10.28	−3.13	0.003
Size (m)	1.55	0.07	1.55	0.06	0.07	0.944	1.72	0.05	1.68	0.06	2.70	0.010
BMI (kg/m^2^)	23.72	3.16	24.38	3.35	−0.99	0.327	22.41	2.53	26.63	2.93	−5.16	≤0.001
WC (cm)	76.02	9.03	77.43	8.56	−0.79	0.429	80.13	8.27	89.79	8.69	−3.86	≤0.001
LDL (mg/dL)	115.65	38.53	100.53	34.29	2.07	0.041	121.92	34.25	103.36	34.21	1.85	0.071
HDL (mg/dL)	46.65	8.56	47.96	10.56	−0.67	0.507	42.63	8.22	45.43	11.34	−0.93	0.356
TG (mg/dL)	95.42	32.56	90.72	40.84	0.62	0.537	90.11	27.56	110.27	48.53	−1.65	0.106
Glucose (mg/dL)	78.30	12.28	77.72	10.00	0.26	0.794	74.47	13.57	81.83	11.37	−2.05	0.046
SBP (mmHg)	105.44	11.10	99.84	11.61	2.43	0.017	106.58	8.88	112.13	9.81	−2.00	0.051
DBP (mmHg)	68.05	7.64	68.35	6.33	−0.22	0.828	71.11	5.99	77.50	8.82	−2.78	0.008
PHC	42.66	4.80	41.45	4.32	1.31	0.192	41.43	3.63	41.76	3.00	−0.35	0.730
MHC	40.72	6.24	42.60	5.06	−1.66	0.100	43.30	3.95	42.64	5.72	0.44	0.665

SD, standard deviation; BMI, Body Mass Index; WC, waist circumference, LDL, low density lipoprotein; HDL, high density lipoprotein; TG, triglycerides, SBP, systolic blood pressure; DBP, diastolic blood pressure; PHC, physical health component of quality of life; MHC, mental health component of quality of life. *P* value. Student's *t*-test (*t*) was used to evaluate the level of difference among the anthropometric, biochemical, and quality of life data and the type of diet and sex. *P* represents the probability that the dietary pattern is differentiated with the mentioned data.

## Data Availability

The data used to support the findings of this study are included within the supplementary information file.

## Supplementary Materials

Supplementary Materials The dataset of our research has been provided as supplementary material.
